# Assessment of error in the MV radiation isocenter position calculated with the Elekta XVI software

**DOI:** 10.1002/acm2.12861

**Published:** 2020-04-02

**Authors:** Jacek M. Chojnowski, George B. Warr, Jonathan R. Sykes, David I. Thwaites

**Affiliations:** ^1^ Mid North Coast Cancer Institute Coffs Harbour Health Campus Coffs Harbour NSW Australia; ^2^ Institute of Medical Physics School of Physics University of Sydney Sydney NSW Australia; ^3^ Department of Radiation Oncology Blacktown Cancer & Haematology Centre Blacktown NSW Australia

**Keywords:** ball‐bearing, Elekta XVI software, EPID, quality assurance, radiation isocenter

## Abstract

The assessment of the coincidence of imaging and radiation isocenters is an important task of regular quality assurance of medical linear accelerators (linacs) as recommended in national and international quality assurance guidelines. A previously reported investigation of the accuracy of the Elekta XVI software to localize the linac radiation isocenter, by comparing statistically with other independent software, has shown some discrepancies at the sub‐mm level. A further investigation is carried out here using a set of reference images and mathematical operations to observe how the Elekta XVI software analyses them. Symmetric mathematical operations on reference images should result in symmetrical outcomes. Three different rotation functions are used in increasing degree of complexity to characterize the Elekta XVI software error in the linac radiation isocenter position. No independent algorithms or phantoms are used in this methodology. The magnitude and direction of the radiation isocenter localization error has been determined to be consistently 0.13 mm or 0.14 mm in the longitudinal direction towards the target depending on the case. The radiation isocenter localization error comprises two separated errors of the Ball Bearing Center by 0.13 mm and MV Field Center by either 0.00 mm or −0.01 mm in the longitudinal direction towards the target. The calculation of the MV Field Center is influenced by the polymethyl methacrylate rod supporting the ball‐bearing. The precise value and the root cause of the error cannot be assessed due to the rounding effect of the results reported by the Elekta XVI software and lack of access to the source code.

## 1 INTRODUCTION

Regular quality assurance of linear accelerators (linacs) is essential in providing accurate and effective radiotherapy. Image guidance systems for tumor localization and patient repositioning have become standard practice. For the image guidance system to be effective, it requires a calibration which correlates the imaging system isocenter and the linac radiation isocenter. Linac manufacturers provide tools and procedures for calibrating proprietary image guidance systems. An important part of regular quality assurance of linear accelerators is to independently verify the coincidence of the imaging and radiation isocenters, as recommended in quality assurance guidelines^1^

Riis et al ^2^ reported that the linac radiation isocenter position determined using the Elekta X‐ray Volume Imaging (XVI) software differs to their independent method, by an average of 0.23 ± 0.13 mm in the longitudinal direction towards the target. This error adds uncertainty to the overall patient setup uncertainty, including registration and table top movement. All such uncertainties should be minimized; however where a systematic error can be identified, it should be corrected. No clear explanation of the cause of this discrepancy was provided, but Riis et al suggested that it might be due to an issue with the Elekta XVI software. In this study, further investigation is undertaken to assess the accuracy of the radiation isocenter position calculated by the Elekta XVI software.

## 2 MATERIALS AND METHODS

### 2.A Linear accelerator and image guidance system

Tests were primarily conducted on the Elekta Versa HD^TM^ linear accelerator (Elekta AB, Stockholm, Sweden) at the Mid North Coast Cancer Institute at Coffs Harbour in NSW, Australia. The linac is equipped with the iViewGT electronic portal imaging system version R3.4.1 and the XVI system version R5.0.4. Secondary comparative tests were carried out on an Elekta Synergy® linac at the same Institute with the same versions of the Elekta iViewGT and XVI software.

### 2.B Radiation isocenter localization procedure

The radiation isocenter localization procedure is part of the kilovoltage (kV) imager flexmap calibration process as described in the Elekta XVI corrective maintenance manual.^3^, ^4^ The kV flexmap calibration process aims to correct clinical kV images for the sag of both the kV EPID panel and kV source due to gravity while the gantry rotates. The correction is stored in the form of a two‐dimensional table (called kV flexmap) mapping the shift that needs to be applied to the kV image in two perpendicular directions at any gantry angle. The kV flexmap calibration process attaches a ball‐bearing phantom to the patient support table and the ball‐bearing is set up to be close to the linac isocenter. Eight megavoltage (MV) images of the ball‐bearing are acquired in total, two at each of the four cardinal gantry angles, using two diametrically opposite collimator angles. The images are analyzed in the Elekta XVI software which calculates the shifts required to be applied to the ball‐bearing position to place it at the linac radiation isocenter. This corrected position is used to further create kV flexmaps for the kV imager panel to ensure the alignment of the kV imaging and MV treatment isocenters.

### 2.C Error estimation methodology

Testing software based on expected output versus controlled input is considered a definitive technique of software validation. To control the input to the Elekta XVI software, simple mathematical operations were applied to an original set of input images.

A set of eight clinical MV images of the ball‐bearing phantom were exported from the Elekta IViewGT software, as Tagged Image File Format (TIFF) files, and imported and processed in Matlab software (The MathWorks, Inc., Natick, USA) using three different methods. Each method used simple MATLAB image processing functions: imread, imrotate and imwrite with a varying number of rotations depending on the method. The processed images were subsequently exported from Matlab software, as TIFF files, and imported back into the Elekta IViewGT software. Clinical, rather than synthesized, images of the ball‐bearing phantom were used to ensure the results are not biased in case the XVI algorithm only works effectively on real images. The kV flexmap calibration process was followed to export the eight MV images from the Elekta IViewGT software and to import them into the Elekta XVI software, in the proprietary (.his) format, where they were analyzed. The results of the analysis in terms of the shifts of the ball‐bearing to the radiation isocenter provided definitive information regarding the assessment of the error in the Elekta XVI software.

This analytical methodology differs from the statistical approach of Riis et al’s work. The Elekta XVI software algorithm is prescriptive and therefore deterministic i.e. the outputs (shifts) are always the same for a given set of the same inputs (images). Thus, statistical methods are not used in the approach presented here.

#### 2.C.1 Method 1

**Fig. 1:**
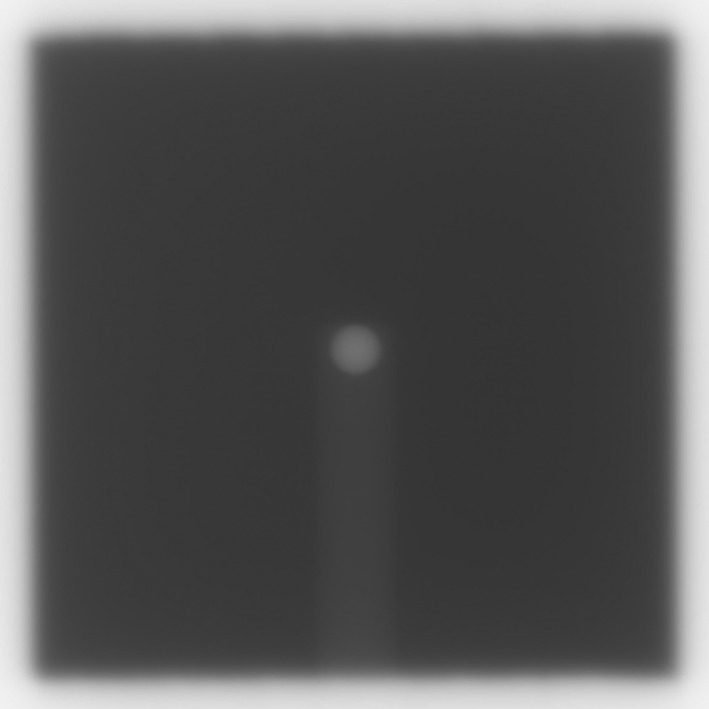
One example of eight original images of the reference ball‐bearing used in the Elekta XVI software.

The first method investigated whether the error in the Elekta XVI software is directly detectable. It is expected that reversing the directions of all original images would result in reversing the recommended shifts of the ball‐bearing compared to the original datasets (see example in Fig. 1). One rotation by 180° was applied to all images.

#### 2.C.2 Method 2

The second method investigated if the polymethyl methacrylate (PMMA) rod supporting the ball‐bearing affects the processing of the images in the Elekta XVI software. Two sets of images were created with directions reversed to each other and with the PMMA rod in the second set oriented perpendicular to the original set, i.e. one set was created by applying a rotation of −90° and the second set with a rotation of +90°.

#### 2.C.3 Method 3

The third method investigated residual shifts of the ball‐bearing from symmetric images. It is expected that symmetric images processed by the Elekta XVI software should result in symmetric shifts of the ball‐bearing, i.e. zero residual shift in each direction.

**Fig. 2:**
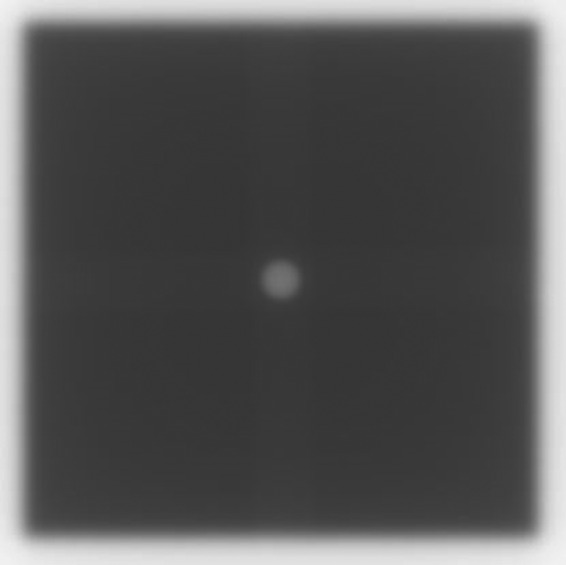
Average of image from Fig 1 rotated by 0°, 90°, 180° and 270° for analysis via method 3.

Symmetric images were created by averaging four sets of images i.e. the original set of images rotated by 0°, 90°, 180° and 270° (see example in Fig. 2 and the MATLAB code in the Appendix A).

#### 2.C.4 Dataset dependency

To check if the results depend on the input dataset, six different input sets of images from different days were considered (five sets using the 6 MV beam and one set using the 15 MV beam).

#### 2.C.5 Linac dependency

To check if the results depend on the specific linac, two additional tests were performed:

- input images from the Elekta Versa HD^TM^ linac were analyzed on a different linac namely an Elekta Synergy® with the same Elekta iViewGT and XVI software version.
- input images from the Elekta Synergy® linac were analyzed on both linacs

## 3 RESULTS AND DISCUSSION

The results from method 1 showed that the Elekta XVI software incorrectly calculated the shift of the reference ball‐bearing to the radiation isocenter by 0.13 mm in the longitudinal direction towards the target. Zero shifts were calculated in the lateral and vertical directions (see Table 1). This outcome confirms the Riis et al suspicion that the Elekta XVI software might cause some discrepancy in the radiation isocenter localization.

**Table 1:** Calculated shift for the ball bearing to the MV radiation isocenter by the Elekta XVI software based on method 1.

Direction	Original images	Original images rotated by 180^o^	Average of original and rotated images	Shift error
	[mm]	[mm]	[mm]	[mm]
Lateral	0.05 B	0.05 A	0	0
Longitudinal	0.58 Target	0.32 Gun	0.26 Target	0.13 Target
Vertical	0.09 Up	0.09 Down	0	0

The results from method 2 showed that the Elekta XVI software calculated the shift of the reference ball‐bearing to the radiation isocenter by 0.135 mm in the longitudinal direction towards the target, a slightly higher value, by 0.005 mm, than observed from method 1. Also the shift in the vertical direction was noted to be changed from zero to 0.005 mm downwards. No change was observed in the lateral direction (see Table 2). The variation of results due to the direction of the PMMA rod is negligible, but nevertheless non‐zero. To further confirm if this error was reproducible another dataset was used and the dependency on the rod direction was not observed. This indicates that results depend, at this small level, on how the Elekta XVI software processes a specific dataset. This is in agreement with Riis et al’s findings that “the phantom asymmetry does not appear to cause the discrepancy”. The possible issue might be due to the sensitivity of the software to the quality of the input images such as noise and contrast, however given the level of effect, this was not further investigated.

**Table 2:** Calculated shift for the ball bearing to the MV radiation isocenter by the Elekta XVI software based on method 2.

Direction	Original images rotated by −90^o^	Original images rotated by + 90^o^	Average of rotated images	Shift error
	[mm]	[mm]	[mm]	[mm]
Lateral	0.45 B	0.45 A	0	0
Longitudinal	0.14 Gun	0.41 Target	0.27 Target	0.135 Target
Vertical	0.14 Down	0.13 Up	0.01 Down	0.005 Down

Method 3 created a symmetric set of images that should result in the Elekta XVI software calculating no shifts in any directions; however the Elekta XVI software calculated the shift of the reference ball‐bearing to the radiation isocenter by 0.14 mm in the longitudinal direction towards the target. Zero shifts were calculated in the lateral and vertical directions (see Table 3). This outcome differs by 0.005–0.01 mm from the results of methods 1 and 2.

**Table 3:** Calculated shift for the ball bearing to the MV radiation isocenter by the Elekta XVI software based on method 3.

Direction	Average of four sets of original images rotated by 0°, 90°, 180° and 270^o^	Shift error
	[mm]	[mm]
Lateral	0.00 B	0
Longitudinal	0.14 Target	0.14 Target
Vertical	0.00 Up	0

**Fig. 3:**
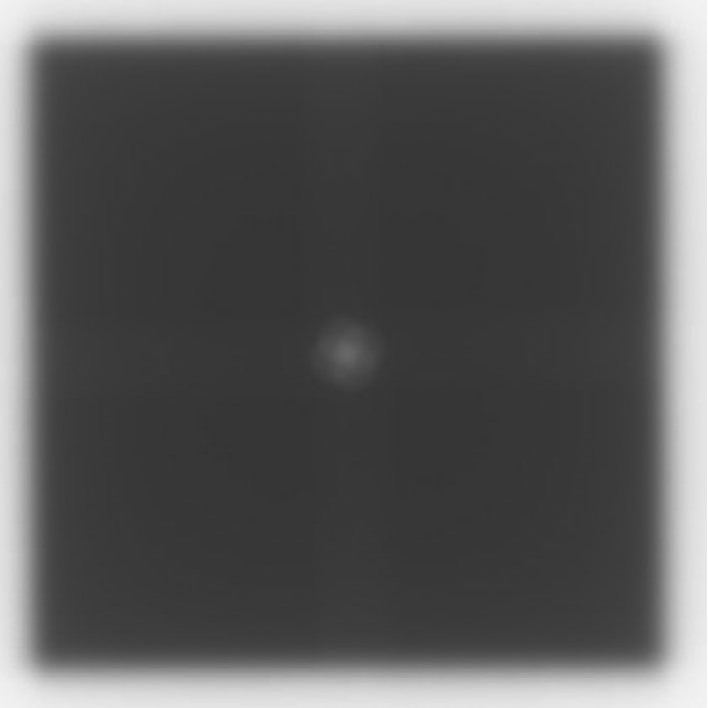
An example of a poor quality averaged image that the Elekta XVI software could not process.

Dataset dependency results, from the method 3 algorithm, are consistent with the method 1 findings that used one particular set of original images. On three occasions the results show the shift of the reference ball‐bearing is 0.13 mm in the longitudinal direction towards the target in each case. On the other three datasets the Elekta XVI software could not process the images. The user cannot modify any pre‐set parameters of the Elekta XVI software and therefore the root cause for not processing the images could not be further investigated. It was only noticed, that those three datasets had overall poor image quality. An example is shown in Fig. 3, where the extensive blurring effect of the ball‐bearing is clearly visible.

The linac dependency test, also using the method 3 algorithm, showed that images analyzed on a different linac (Elekta Synergy®) give exactly the same results for one particular set of original images (0.14 mm). This lack of linac dependency was expected, since the software versions are the same on both linacs. The analysis of the input images from the Elekta Synergy® on the Elekta Versa HD^TM^ linac (reversed scenario) showed that the error in the shift of the reference ball‐bearing is equal to 0.13 mm in the longitudinal direction towards the target. The result was the same when input images from the Elekta Synergy® were analyzed on the same linac, as expected. This indicates that the results are dependent on the dataset, not the linac itself. As before, the small variations are likely to be due to the sensitivity of the software to the quality of the input images such as noise and contrast.

The numerical shift value of the ball‐bearing is limited to two decimal places of a millimeter, thus the assessment of the Elekta XVI software is limited to this rounding effect. Determination of the shift with higher precision is not possible in a clinical testing environment.

It is concluded that the systematic error in the localization of the radiation isocenter (0.13 mm or 0.14 mm) is caused by an issue in the Elekta XVI software. The results are within the estimation reported by Riis et al of 0.23 ± 0.13 mm, however the mean value there was almost double the shift found in this work. This could be attributed to the fact that Riis et al reported the statistical difference between the Elekta XVI software and independent software using different algorithms, whereas here only the Elekta XVI software has been used throughout for analytical scrutiny to minimize variables. While Riis et al could not clearly identify a cause of their observed differences, they suggested a systematic issue in the XVI software was the likely cause and that this proprietary software should be improved.

Further analysis of the method 3 results revealed an interesting Elekta XVI software feature that otherwise would not be possible to see in a clinical scenario. The shift of the reference ball‐bearing is calculated as the difference between the Ball Bearing Center and the MV Field Center. Those values are reported by the Elekta XVI software. It was observed that the Y value (corresponding to longitudinal direction) of the Ball Bearing Center was always 0.13 mm. This effect could be attributed to a small algorithm error of the ball‐bearing by half of a pixel (i.e. 0.5*0.25 mm = 0.125 mm rounded to 0.13 mm). The MV Field Center was always either 0.00 mm or –0.01 mm for all input images using method 3 (see Table 4). This effect could be attributed to the fact that the region of the image with the open radiation field has more pixels and lower contrast compared to the region of the image with the ball‐bearing and therefore is more sensitive to the quality of the input images. The observation of the MV Field Center results combined with the conclusion from method 2, that there is a directional rod dependence of 0.005 m, allowed an explanation of why results from method 3 are either 0.13 mm or 0.14 mm. Depending on whether the Elekta XVI software is sensitive to the rod for a particular dataset, the detection of the MV Field Center is either 0 mm or −0.01 mm. This value combines with the Ball Bearing Center value of 0.013 mm to determine the overall shift of the ball‐bearing to the radiation isocenter as 0.13 mm or 0.14 mm (see Table 4). The absolute value of the MV Field Center of 0 mm or 0.01 mm is double the value of the rod effect of 0 mm or 0.005 mm. The reason for that is not exactly clear, but could be simply attributed to the rounding effect of the reported shift to two decimal places. The overall error in the localization of the radiation isocenter is considered clinically insignificant.

**Table 4:** The average center of all images in Elekta (world) coordinates relative to the panel center.

Coordinate	MV Field Center	Ball Bearing Center	Difference
	[mm]	[mm]	[mm]
X	0.00		0
Y	0.00 or −0.01	0.13	0.13 or 0.14
Z	0.00		0

Riis et al investigated the influence of the MLC orientation on the radiation isocenter localization and could not observe any correlations. MLC orientation dependency was not specifically examined in this study since in our opinion this effect (similar to MLC leaf miscalibration) is eliminated in the radiation isocenter localization procedure by acquiring ball‐bearing images with two opposite collimator angles.

## 4 CONCLUSION

It has been determined that the Elekta XVI software used to define kV flexmaps for the image guidance system incorrectly calculates the MV radiation isocenter position by either 0.13 mm or 0.14 mm in the longitudinal direction towards the target. This is caused by an incorrect calculation of both the Ball Bearing Center by 0.13 mm and MV Field Center by 0.00 mm or −0.01 mm in the longitudinal direction towards the target. The incorrect calculation of the MV Field Center is influenced by the PMMA rod supporting the ball‐bearing. The error in the Elekta XVI software is clinically insignificant, however our opinion is the same as Riis et al that the Elekta XVI software should be improved to achieve better accuracy in the linac radiation isocenter localization.

## CONFLICT OF INTEREST

The authors declare no conflict of interest.

## Appendix A

The MATLAB code for the method 3.

for i = 1:8

% reading 8 input images 1.tiff, 2.tiff…

file_name = strcat(num2str(i),'.tiff');

img = imread(file_name);

% rotating input images by 0, 90, 180 and 270 degrees

img0 = img;

img1 = imrotate(img,90);

img2 = imrotate(img,180);

img3 = imrotate(img,270);

% averaging all 4 images

img=(double(img0)+double(img1)+double(img2)+double(img3))/4;

img = uint16(img);

% saving output images with the underscore _1.tiff, _2.tiff…

file_name = strcat('_',file_name);

imwrite(img,file_name);

end
